# Middle Ear Cholesteatoma and Vestibular Schwannoma Resection Followed by Cochlear Implant: Surgical Challenges and Audiological Outcomes

**DOI:** 10.3390/jcm12227139

**Published:** 2023-11-17

**Authors:** Natalia Carasek, Danielle Cristovão, Lucas Alves Teixeira Oliveira, Fernanda Ferreira Caldas, Fernando Massa Correia, Thais Gomes Abrahão Elias, Rivadávio Amorim, Fayez Bahmad Jr

**Affiliations:** 1 Faculty of Health Sciences, University of Brasília, Federal District, Brasilia 70910-900, Brazil; ncarasek@gmail.com (N.C.); fgadanielle.c@gmail.com (D.C.); lucasat.oliveira@gmail.com (L.A.T.O.); 2 Brazilian Institute of Otorhinolaryngology, Federal District, Brasília 70710-149, Brazil; 3 Faculty of Medical Sciences, University of Brasília, Federal District, Brasilia 70910-900, Brazil

**Keywords:** vestibular schwannoma, acoustic neuroma, middle ear cholesteatoma, cochlear implants, unilateral hearing loss, translabyrinthine approach

## Abstract

(1) Background: The occurrence of vestibular schwannoma (VS) associated with cholesteatoma is rare. A hearing impairment is one of the most significant issues in such cases. Moreover, the presence of middle and inner ear pathologies combined may represent a surgical challenge. No studies have described a combined surgical approach for these coexisting conditions (VS and cholesteatoma), nor the hearing rehabilitation outcomes of using cochlear implants for these patients. (2) Case Report: This paper is on a female patient who underwent simultaneous surgical treatments for VS and middle ear cholesteatoma in the right ear followed by a cochlear implant, describing the technique and the audiological results. (3) Conclusions: The surgical approach was successful and enabled the resection of lesions with the auditory nerve and cochlea preservation. Cochlear implantation in the right ear showed positive postoperative results, with an improvement in the results with the CI in silent and noisy environments.

## 1. Introduction

Vestibular schwannoma (VS) is a benign tumor of the Schwann cells from the vestibulocochlear nerve, which occurs at the cerebellopontine angle (CPA) and/or internal auditory canal (IAC) [1,2]. Its most common symptoms are progressive sensorineural hearing loss, vertigo and tinnitus. The treatment options vary according to the location, size of tumor and preoperative hearing thresholds [2]. After diagnosis, the treatment options available for VS are observation, stereotactic radiation therapy, complete surgical excision and subtotal resection with planned radiation therapy [3]. The surgical approach has three main techniques to accessing the tumor: translabyrinthine, middle fossa or retrosigmoid approaches. They each have advantages and disadvantages [3]. Nevertheless, regardless of the tumor management strategy and cochlear nerve status, many patients eventually lose their hearing during the disease course [4,5].

Recent studies have shown that cochlear implants (CI) can function in the presence of retrocochlear pathologies, such as vestibular schwannoma [1,5,6,7,8]. The traditional hearing device developed for these cases is the Auditory Brainstem Implant (ABI), that requires a technically challenging and potentially riskier surgery, with poorer audiological outcomes compared to those of CIs [9]. In cases of VS resection, cochlear implantation is only possible if the fibers of the cochlear nerve and the cochlea are preserved during the posterior labyrinthectomy and IAC tumor removal [5]. The previous standard strategies for hearing rehabilitation in Single-Sided Deafness (SSD) caused by vestibular schwannoma were either a Contralateral Routing of Signal (CROS) hearing aid or a Bone Conduction Device (BCD). Both options could provide sound awareness for the deaf side, but they cannot reproduce the audiological benefits of binaural hearing achieved through CIs [5].

Middle ear cholesteatoma is another type of ear disease that can also manifest with hearing loss. The main symptoms are otorrhea, tinnitus and conductive or mixed progressive hearing loss [10]. It is a non-neoplastic epithelial lesion that contains layers of keratin in a cavity lined by keratinizing squamous epithelium and subepithelial connective tissue. Despite its benign nature, a cholesteatoma can cause serious complications by eroding nearby structures or precipitating an infection. The surgical removal of the lesion is considered the only effective therapy [11]. The postoperative audiological outcomes vary according to the size of the lesion and reconstructive strategies used for conductive loss. In cases of severe or profound hearing loss, the rehabilitation options also include CIs [10].

The presence of a middle ear or IAC pathology may represent a challenge to performing CI surgery. When treating Cholesteatomatous Otitis Media, the technique of a subtotal petrosectomy associated with CIs provides protection against infection and the extrusion of the device [12]. It is a meticulous procedure, with a high technical difficulty level.

A few articles describe the co-occurrence of vestibular schwannoma and cholesteatoma in the same patient. The unilateral manifestation of both diseases at the same time is even more rare [13,14,15]. To our knowledge, there are no studies published so far describing this combined surgical approach nor the hearing rehabilitation outcomes in CI for patients with the coexistence of these pathologies. Therefore, we present a case of a female patient who underwent simultaneous surgical treatment for VS, middle ear cholesteatoma, and auditory rehabilitation with cochlear implants, including a detailed follow-up of the audiological results.

## 2. Case Report

**Figure 1:**
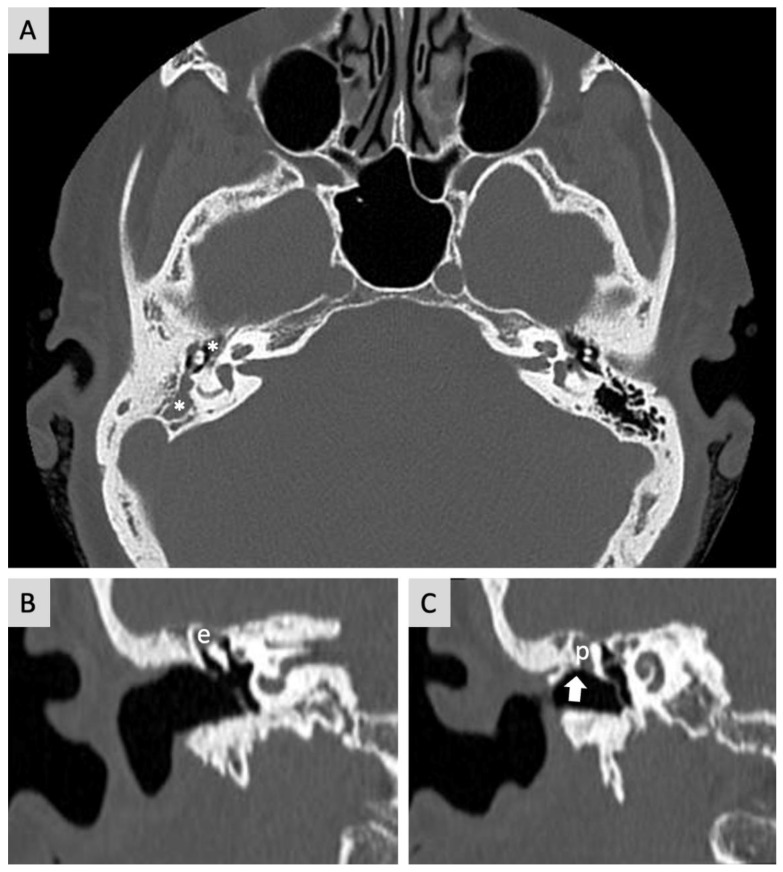
Head CT. (**A**) Axial—opacification of mastoid cells and middle ear on the right side (*). (**B**,**C**) Coronal—filling of the epitympanum (e) and Proussak space (p) with erosion of the spur of Chaussé (arrow) in the right ear.

A 54-year-old female was referred to the Department of Otorhinolaryngology complaining of progressive hypoacusis in the right ear for the last 19 years. No previous history of diseases was reported. A physical examination revealed right attic tympanic membrane perforation. Computed Tomography (CT) showed the opacification of mastoid cells and the epitympanum, suggesting cholesteatomatous chronic otitis media in the right ear (Figure 1). Magnetic Resonance Imaging (MRI) also enabled the identification of a lesion in the right IAC measuring 1.5 × 0.8 × 0.8 cm, resembling VS.

**Figure 2:**
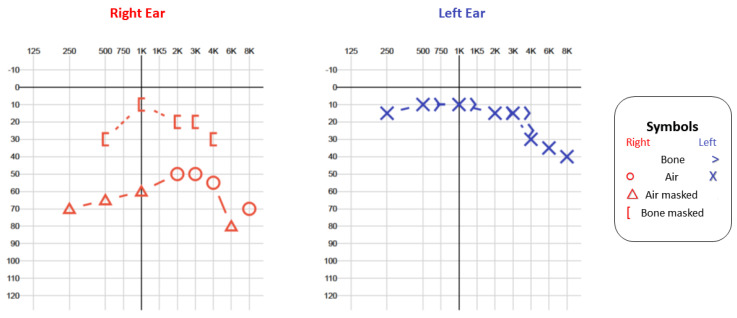
Pure tone audiometry.

**Figure 3:**
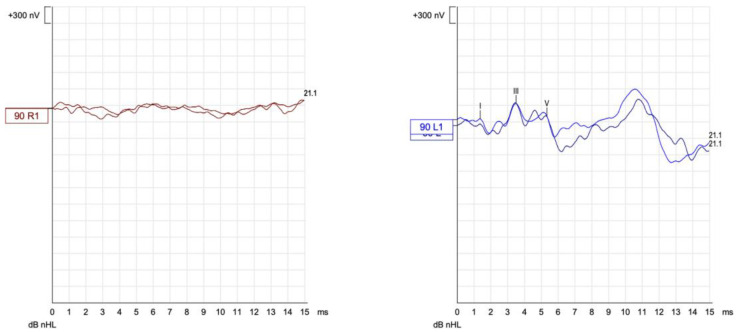
Auditory Brainstem Response (ABR) with click 90 dB stimuli showed no response—absence of waves—in the right ear (red) and normal responses—presence of waves I, III and V with normal latencies and interpeak intervals—in the left ear (blue).

Initial audiological evaluation with pure tone audiometry (PTA) showed conductive hearing loss in the right ear with a tritone average of 56 dB, and the left ear had normal thresholds. The Right Auditory Brainstem Response (ABR) had waves I, III and V present, with increased absolute latencies and normal inter-peak intervals, which are suggestive of conductive loss. The patient avoided surgery on multiple occasions, was lost during the follow-up and returned after 2 years for reassessment due to a vertiginous crisis. At this point, the patient presented with mixed hearing loss, and an important difficulty in comprehension in noise. Although the PTA had a tritone average of 58 dB, the air–bone gaps had increased considerably (Figure 2), and the ABR showed no response in the right ear (Figure 3).

**Figure 4:**
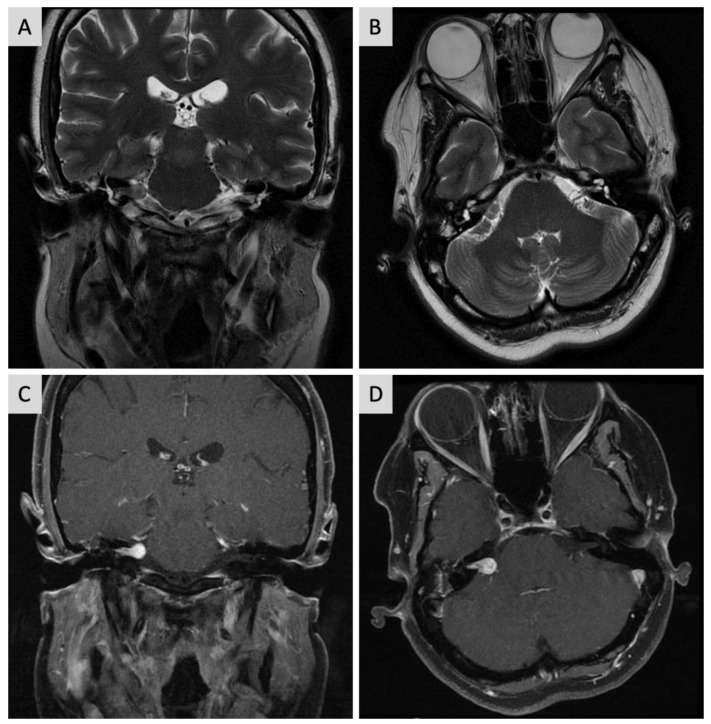
Cranial MRI showing lesion in the right cerebellopontine angle (**A**) T2-weighted coronal image. (**B**) T2-weighted axial. (**C**) T1 contrast-enhanced coronal. (**D**) T1 contrast-enhanced axial.

New MRI indicated an increase in the dimensions of the expansive lesion inside the IAC, at this point measuring 2 × 1.3 cm, in contact with the cochlear modiolus. It extended and partially obliterated the cerebellopontine angle cistern and brainstem, also compressing the emergence of the right vagus nerve, but with no change in the brainstem signal (Figure 4).

**Figure 5:**
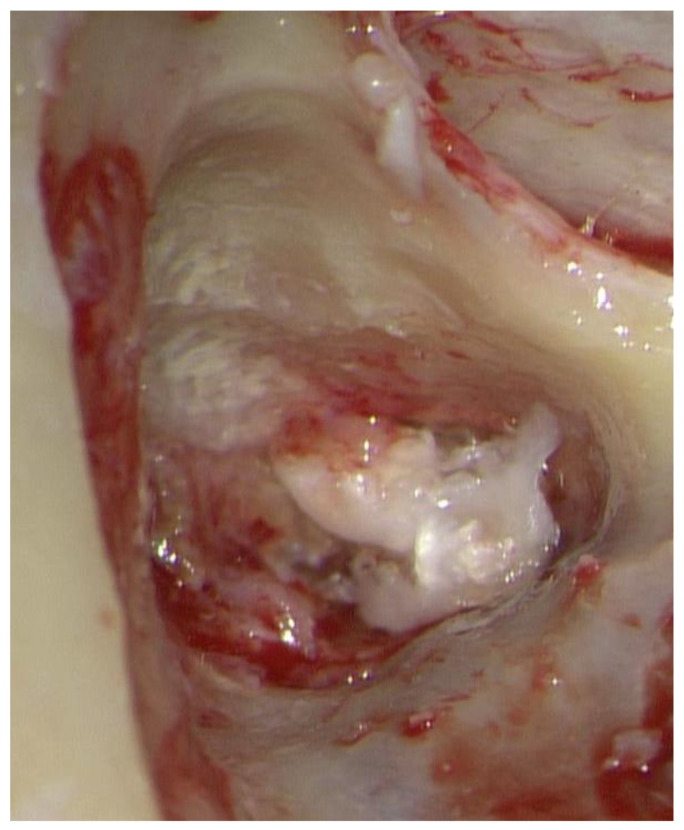
Tympanomastoidectomy revealed cholesteatoma lamellae in the aditus ad antrum.

**Figure 6:**
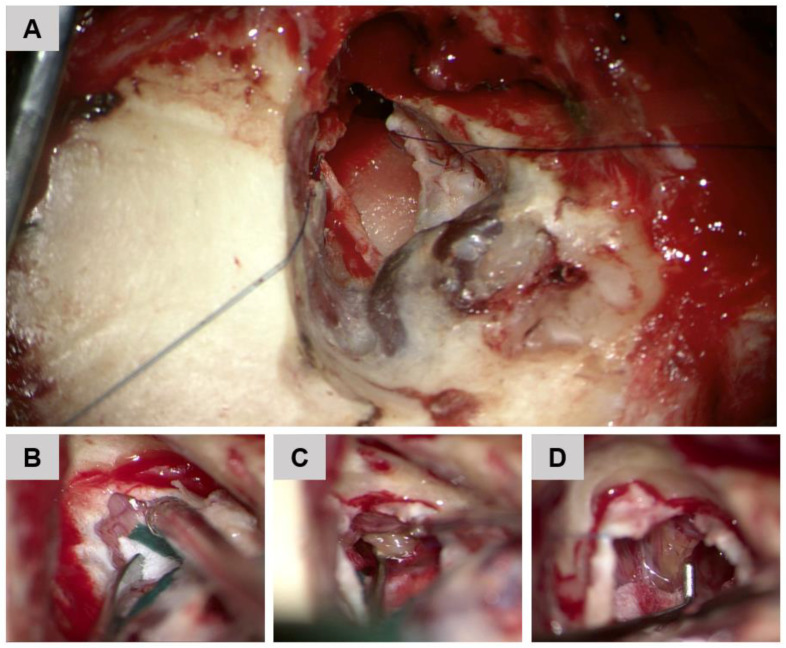
(**A**) Translabyrinthine access to the cerebello-pontine angle. (**B**) Opening of the internal auditory canal. (**C**,**D**) Resection of the vestibular schwannoma in the internal auditory canal.

**Figure 7:**
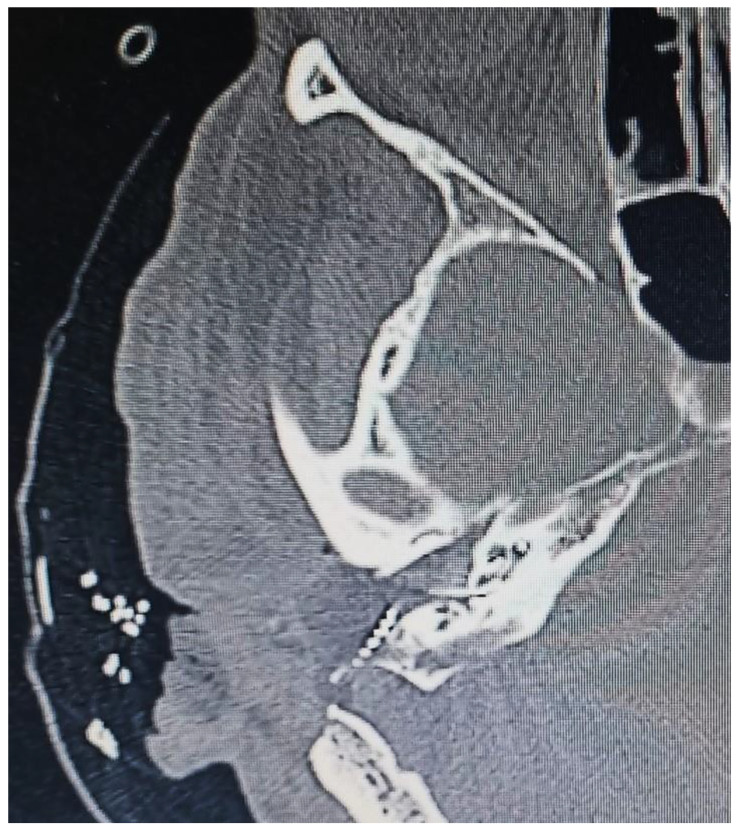
Postoperative Axial Head CT revealing correct electrode array insertion in the right cochlea.

The patient underwent tympanomastoidectomy with the resection of the cholesteatoma (Figure 5) and subtotal petrosectomy with blind sac ear canal closure, followed by the translabyrinthine approach to treating the vestibular schwannoma (Figure 6). The preservation of the cochlea and auditory nerve allowed subsequent cochlear implantation in the right ear. Complete electrode insertion was performed via round window, with normal intraoperative impedances. Control Head CT on the first day after surgery showed no evidence of pneumocephalus or hemorrhaging and certified the correct electrode insertion (Figure 7).

**Figure 8:**
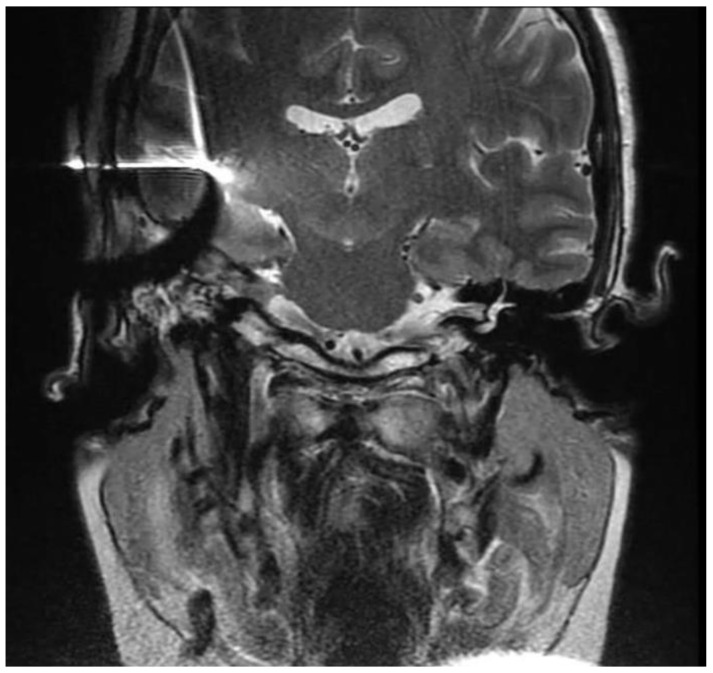
Postoperative MRI.

The follow-up otoscopy revealed blind sac ear canal with a wound in great condition. The patient is currently undergoing weekly aural rehabilitation therapy with a team specialized in cochlear implants. Despite magnetic artifacts due to the cochlear implant in the right ear, postoperative MRI (Figure 8) allowed the identification of heterogeneous material filling the surgical cavity, corresponding to the scarred area, and the T2-weighted scan revealed hyposignal in the right cochlear basal turn due to the cochlear implant electrode array. Periodic MRI scans will be performed to monitor possible neuroma recurrences.

**Figure 9:**
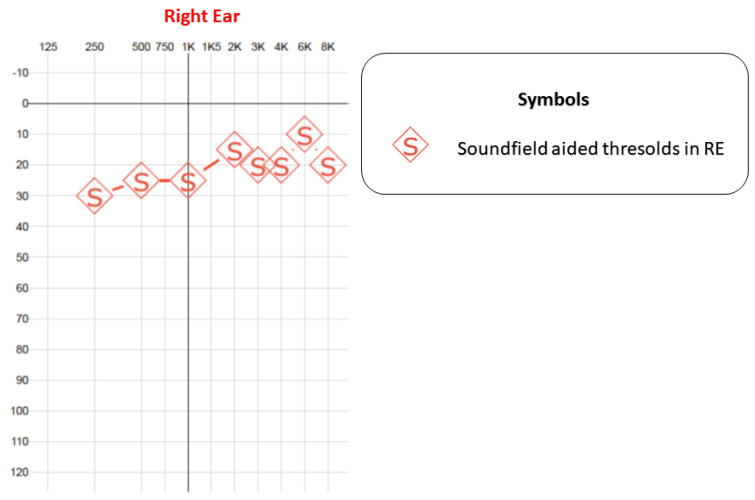
Postoperative aided hearing thresholds with CI on the right ear. Measurement obtained in soundfield with contralateral masking. RE—right ear.

The 6-months postoperative PTA-aided results demonstrated a tritone average of 26 dB in the right ear, with contralateral masking (Figure 9). The Brazilian Hearing in Noise Test (HINT), which measures the difficulty of understanding speech in silent and in noisy environments, was performed along with a Visual Analog Scale (VAS) test to measure the subjective listening effort. The results are shown in Table 1. After only a few sections of vestibular rehabilitation therapy, the patient’s dizziness was resolved. 

## 3. Discussion

The presence of VS and middle ear cholesteatoma in the same patient represents a surgical challenge. The disease courses and the combined surgical approach are both unlikely to preserve hearing. Aiming to surgically remove both lesions and rehabilitate the patient’s hearing, our team of surgeons had to adapt the classical technique. Thus, the resection of cholesteatoma and a subtotal petrosectomy were successfully performed, followed by the enlarged translabyrinthine approach to treating the neuroma. In addition, the cochlea and auditory nerve were preserved, and subsequent cochlear implantation could be carried out in the right ear.

Before the surgical intervention, the patient had moderately severe mixed hearing loss in the right ear and normal tritonal average in the left ear, according to Lloyd and Kaplan [16]. The postoperative expected results place this patient in the SSD category. For these patients, although CROS and BCD can provide sound awareness on the impaired side, they cannot reproduce the audiological benefits of binaural hearing, which are achieved only via cochlear implantation [5]. Among those benefits, the head shadow effect, the binaural summation and the binaural squelch are highlighted. 

Each of the two ears substantially contributes to the action potentials that reach the brainstem, which are referred to as binaural loudness. In a binaural-hearing subject, the number of action potentials may double when both ears are used instead of one. The processing of information is also more sensitive to small differences because the just-noticeable differences in intensity and frequency improve with signal redundancy. Therefore, speech recognition in noisy environments is improved [17]. When speech and noise are spatially separated, there is an improvement in the signal-to-noise ratio (SNR) due to the acoustic shadow provided by the head, benefiting the side with a better SNR or the one closest to the signal. The head shadow effect not only is useful in noisy environments, but also improves sound localization. Binaural summation occurs when both ears receive the same signal; the central auditory cortex compares monoaural to binaural cues, improving the perception of loudness. The binaural squelch is the integration of signal that comes in the two ears at central auditory cortex, comparing the different SNRs and highlighting the signal of interest. Consequently, it provides a perceived enhancement of sound in the presence of background noise [18,19,20]. 

A recent systematic review [21] on the simultaneous skull base and CI approaches demonstrated that most subjects achieved open-set comprehension and subjective benefits from CIs. In another retrospective study [22], the subjects achieved a level of speech understanding higher than 50% at 65 dB. In accordance with these literature findings, our subject achieved 75.42% during silence in the implanted ear. The patient did not achieve a negative SNR, but reported less subjective auditory effort, which can be considered a success due to the short time post activation.

One of the patient’s preoperative complaints was the difficulty of comprehension in noisy environments. After only six months postoperatively, she achieved around 64.50% hearing in fixed-noise environments with the CI ear and 99% with bilateral hearing (NH + CI). Our results support the findings of a recently published randomized controlled trial [23] that showed a larger improvement in speech perception in noisy environments with cochlear implantation for SSD when compared with those of CROS and BCD. During the evaluation and decision making about which hearing device to use, it is important to advise the patient about the benefits of each and to consider the primary complaint, providing better outcomes and an increased quality of life.

Another question that could be raised is about whether it is better to perform the procedures in sequence or concomitantly. When treating patients with SSD, it is important to evaluate the status of the contralateral ear; if there is a risk of, or an emerging hearing loss in the other ear, the surgeon tends to opt for concomitant auditory rehabilitation. In our case, although the contralateral ear had a normal tritonal average, there was a lowering of the thresholds at high frequencies that necessitated a follow-up.

In addition, some other factors should be taken into consideration: (i) the duration of deafness is intimately related to speech perception outcomes, so delaying the rehabilitation may jeopardize the results [5]; (ii) the secondary cochlear fibrosis occurrence rate following translabyrinthine surgery may be as high as 46% [5]; therefore, the sequential approach may be hampered by fibrosis. An MRI-based study with patients before and after the translabyrinthine removal of vestibular schwannoma [24] showed evidence of mild fluid signal changes inside the cochlea 6 months postoperatively, suggesting the onset of fibrotic tissue. At 48 months, it had progressed to severe involvement. Given the early onset of cochlear fluid signal changes on T2MRI, and the progression of these changes on subsequent images, cochlear implantation should ideally be performed either simultaneously with translabyrinthine surgery or as an early second-stage procedure to maximize the chance of successful electrode insertion. Considering these factors, we opted to perform both resections and the cochlear implantation at the same time.

An alternative approach to this case would be to access the VS through the middle fossa or retrosigmoid approach in a first stage and, subsequently, address the cholesteatoma through tympanomastoidectomy. In an optimistic scenario, this sequence could allow reasonable hearing preservation, enabling the patient to use hearing aids in the right ear, instead of CI. Even though this was a viable option in this case, the patient had an active role in this decision. Surgery in two stages was offered to the patient, both via the middle fossa and retrosigmoid access points.

The advantages of translabyrinthine access are consistent facial nerve identification, no tumor size limitation, no intradural drilling, the wide exposure of the posterior fossa and low recurrence and headache rates. The disadvantages include complete hearing loss and the need for an abdominal fat graft [3]. The middle fossa provides better hearing preservation rates, it does not need intradural drilling and causes low rates of headaches. However, it is more appropriate for smaller tumors because of its limited exposure of the posterior fossa and requires temporal lobe retraction [3]. The retrosigmoid approach has no tumor size limitation, provides wide exposure, and hearing preservation is possible. Nevertheless, this option provides limited exposure of the lateral IAC, requires intradural drilling and cerebellar retraction, may cause postoperative headaches, and facial nerve identification occurs relatively late in the dissection [3].

After being thoroughly informed of the risks and advantages of each of each approach, and since the patient did not want to undergo a two-stage procedure, translabyrinthine access was chosen. Furthermore, we pondered whether the preservation of hearing in non-translabyrinthine surgery can be temporary, as some of these patients have long-term hearing degeneration after the surgical manipulation of the internal auditory canal [3]. The mechanisms of long-term hearing loss are unclear. Some suggest microscopic tumor recurrence, the development of endolymphatic hydrops, or toxicity due to the use of muscle in packing the IAC as some of the factors for the decline [25].

Another point worth mentioning is that by the time of the surgery, the patient was already suffering from dizziness (her main complaint). Although, the labyrinthectomy via translabyrinthine access leads initially to vertigo and requires subsequent balance rehabilitation, it is also the surgical procedure of the temporal bone used to treat intractable and refractory vertigo [26]. This procedure surgically removes the neuroepithelial elements of the semicircular canals and vestibule, causing the ablation of abnormal signals from a diseased vestibular system to facilitate central compensation [24]. Postoperatively, after only a few sessions of vestibular rehabilitation therapy, the patients’ dizziness was resolved.

Even though this procedure via the translabyrinthine approach with simultaneous cholesteatoma removal and cochlear implantation was feasible and reached optimal results, we are describing a very specific situation—concomitant middle ear cholesteatoma and VS. The goal of this report is not to attest that this combined approach is the best strategy, but to report a successful path for future reference. Surgeons must individualize their conduct for each case and never underestimate the inherent risks of each procedure.

## 4. Conclusions

The presence of vestibular schwannoma and middle ear cholesteatoma in the same patient represents a surgical challenge. For such clinical circumstances, the classical technique needed to be adapted to optimize the treatment and enable successful outcomes. We described a case, illustrating how it is possible to perform, at the same time and with safety, the resection of both lesions and cochlear implantation in the same ear and during the same surgical session, demonstrating the successful audiological results that followed.

## Figures and Tables

**Table 1:** Hearing in noise test results. CI corresponds to aided responses with CI only (contralateral masking was used). Adaptive noise results are demonstrated with signal-to-noise ratio (SNR) thresholds. The visual analogue scale corresponds to subjective listening effort measure. The lower the value is, the lower the perception of the subject’s effort in the task is.

Test Condition	Results	Visual Analogue Scale
SILENCE 65 dB	CI: 75.42%	3
FIXED NOISE 55 dB SNR: +10 dB	CI: 64.50%	4
ADAPTIVE NOISE 55 dB	CI: +3.5 dB	4

## Data Availability

Data are contained within the article or Supplementary Materials.

## Supplementary Materials

The following supporting information can be downloaded at: https://drive.google.com/file/d/1hKOzYyBFJJc9fRzkWr-mmAwnGezXzxJ-/view?pli=1 (accessed on 17 September 2023), Video S1: Complete surgery video.
